# Comprehensive Quality and Bioactive Constituent Analysis of Celery Juice Made from Different Cultivars

**DOI:** 10.3390/foods11182719

**Published:** 2022-09-06

**Authors:** Jun Yan, Xiaofeng Yang, Lizhong He, Zhiwu Huang, Mingfen Zhu, Linhua Fan, Han Li, Lingyun Wu, Li Yu, Weimin Zhu

**Affiliations:** 1 Horticulture Research Institute, Shanghai Academy of Agricultural Sciences, Key Laboratory of Protected Horticulture Technology, Shanghai 201403, China; 2 Zhuanghang Integrated Experimental Station, Shanghai Academy of Agricultural Sciences, Shanghai 201415, China; 3 Organ Management Office, Shanghai Academy of Agricultural Sciences, Shanghai 201403, China

**Keywords:** celery juice, basic properties, secondary metabolites, volatile compounds

## Abstract

Celery juice is rich in bioactive constituents, has good health properties, and is becoming much more popular, with its demand continuing to rise. The results of this study show that celery juice from Chinese cultivars contains more bioactive constituents, whereas celery cultivars from the United States and European countries have a higher juice yield. Compared with the other juices, the juices of five cultivars may taste sweeter, and the juices of three cultivars had a higher antioxidant capacity. The juices of six cultivars (three with the highest antioxidant capacity and three with the lowest antioxidant capacity) were selected to analyze bioactive constituents by LC/MS and GC/MS. A total of 71 phenolic acids, 38 flavonoids, 18 coumarins, 41 terpenoids, and 11 phthalides were detected in the juices of the six celery cultivars. The contents of 14 compounds had a more than 10-fold difference among these celery juices. This study first evaluated the comprehensive quality of the juices made from 26 celery cultivars and then analyzed the differences in bioactive constituents in the juices of6 celery cultivars. These findings provide information for the further study on the health functions of celery juice and can also guide celery juice production and celery breeding.

## 1. Introduction

Celery is a vegetable from the Apiaceae family; it originated in the Mediterranean Basin and the Middle East and is widely cultivated worldwide. Celery varieties from China have thin and hollow petioles, whereas celery varieties from Europe and the United States have thick and solid petioles. Celery varieties are classified into four types (red, green, white, and yellow) according to the petiole color. Celery is rich in various beneficial bioactive constituents, including flavonoids, phenolic acids, furocoumarins, terpenoids, and phthalides [1]. Phthalides contribute to the characteristic celery odor [2]. Celery has antioxidant, anti-inflammatory, anticancer, antirheumatic, anti-hypertension, antidiabetic, and neuroprotective properties [3]. The variety can influence the content of bioactive constituents in the plant [4]. Therefore, differences in the bioactive constituent content of different celery varieties are expected.

Celery was first used as a medicinal crop. After a long period of domestication and breeding, it is now used as a vegetable in cooking. Celery is usually fried with other ingredients or used as a condiment to increase soup flavor, and it can be consumed raw, mainly as a salad or as celery juice. It has been reported that celery subjected to thermal processing has a decreased antioxidant activity of phenolic compounds, and their structure is converted into one that is not absorbed and metabolized in the human body [5]. Raw vegetables retain their nutrients in large amounts. Lasria et al. [6] found that, compared to boiled celery water, celery juice is more effective in reducing hypertension. Celery juice can either be consumed alone or mixed with other fruit and vegetable juices. It has become a health beverage in people’s daily lives.

It was demonstrated that celery juice can be used as a supplement in the treatment of hypertension [7]. Kolarovic et al. [8] reported that celery juice can protect patients receiving doxorubicin therapy from congestive heart failure in the treatment of various cancers. The high content of phenolics enhances the health benefits of the juice but aggravates its astringency, which is not welcomed by some consumers [9]. To date, most studies have focused on the process method and pharmacological functions of celery juice made from Western celery, while the comprehensive quality of the juice from different celery varieties is unclear. The purpose of this study was to evaluate the juice quality of 26 celery cultivars and then analyze the differences in secondary metabolites and volatile flavor compounds in the juices of different celery cultivars to provide information for the further study on the health functions of celery juice and to guide celery juice production and celery breeding.

## 2. Materials and Methods

### 2.1. Plant Material and Juice

**Figure 1:**
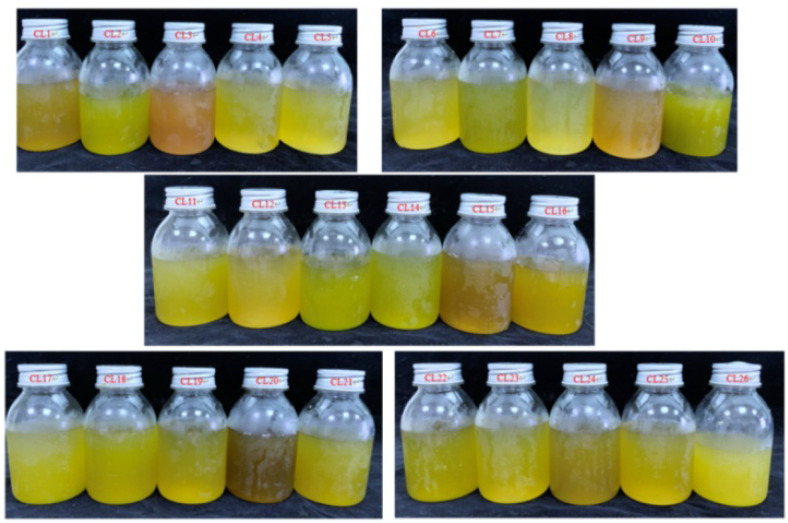
The pictures of celery juice made from 26 cultivars.

A total of 26 celery cultivars (Table 1) collected by our laboratory staff were selected for this study. The 26 cultivars were sown in a greenhouse in Zhuanghang Experimental Station of the Shanghai Academy of Agricultural Sciences in September 2020. Each cultivar was planted in three cultivation troughs containing peat: perlite (3:1, *v*/*v*) using a random block group design; each cultivation trough measured three square meters. All cultivation troughs were subjected to the same cultivation and management conditions. The greenhouse condition and agricultural technology were the same as our previous report [10]. Each cultivar was harvested at its corresponding harvest period. Five plants were randomly selected from each plot. A total of 15 plants of each cultivar were used to determine the indexes after juice preparation. The petioles were washed and squeezed using an Omega J8228 slow juicer (Harrisburg, PA, USA) (Figure 1), and the juice was stored at −80 °C until further analysis.

### 2.2. Physical Properties

After harvesting, the weight of each plant was determined using an electronic balance (±0.01 g sensitivity). The juice yield was determined using the ratio of juice weight (g) to raw material weight (g). The transmittance of the juice at 680 nm (T680) was measured with a UV16A spectrophotometer (Shimadzu, Kyoto, Japan). Ten milliliters of juice were poured into a colorless and transparent Petri dish for color assessment using a CR-400 colorimeter (Minolta, Tokyo, Japan). After calibrating with a standard white tile, the instrument measured the juice color using a D65 light source and 0° observation angle. The parallel measurement of each sample was performed three times. The juice color was characterized by three indicators (L*, a*, and b* values). The L* value represents the brightness from 0 (black) to 100 (white). The a* value represents red (+) and green (-), and the b* value represents yellow (+) and blue (-).

### 2.3. Basic Nutritional Components

The soluble solid content (SSC) was determined using a portable digital refractometer (Atago PR-101a; Tokyo, Japan). The soluble sugar content was determined through anthrone-sulfuric acid colorimetry. The juice (2 mL) was centrifuged at 12,000 rpm for 20 min. The supernatant (0.5 mL) was placed into 15 mL centrifuge tubes, and distilled water (1.5 mL), anthrone ethyl acetate solution (0.5 mL, 1 g anthrone dissolved in 50 mL ethyl acetate), and concentrated sulfuric acid (5 mL) were added. The mixture was heated at 95 °C for 3 min and cooled in ice water to room temperature. The absorbance was determined with a spectrophotometer (Shimadzu UV-1800, Kyoto, Japan) at 630 nm. The same procedure was used for the standard solutions of glucose to draw a standard curve. The soluble sugar content of the juice was calculated based on the standard curve.

The soluble dietary fiber (SDF) of the juice was determined using a kit (K-TDFR 200; Megazyme, Ireland) based on the AOAC International Official Methods (AOAC, 991.43, 2005). The samples were freeze-dried under vacuum, transferred to MES-Tris buffer, and digested with three enzymes (protease, a-amylase, and amyloglucosidase). The enzymatic hydrolysate was precipitated with ethanol; the precipitate was washed with 95% ethanol and dialyzed using a 200 Da dialysis bag, and then dried in vacuum to obtain the SDF. The SDF content (g/100 g) was weighed after the removal of the protein and ash.

### 2.4. Antioxidant Properties

The total phenolic compounds in the celery juice were determined using the Folin–Ciocalteu method as described by Obeng et al. [11], with some modifications. The juice (0.3 mL) was mixed with 1.5 mL Folin–Ciocalteu (0.2 mol/L) solution for 5 min, and then 1.2 mL sodium carbonate (7 g/100 mL) solution was added to the mixture, which was left to stand at 24 °C for 1 h. The absorbance of the reactant was measured with a spectrophotometer (Shimadzu UV-1800, Kyoto, Japan) at 765 nm. The total phenolic content of the juice was expressed as mg/L gallic acid equivalent.

The total flavonoid content in the celery juice was determined using the aluminum chloride method described by Hmidani et al. [12], with some modifications. The juice (0.5 mL), 2 mL distilled water, and 0.15 mL NaNO_2_ (0.05 g/mL) were mixed and left to stand at 24 °C for 5 min; then, 0.15 mL AlCl_3_ (0.1 g/mL) was added to the mixture, which was left to react at 24 °C for 6 min and subsequently mixed with 1 mL NaOH (1 mol/L) and 1.2 mL distilled water. The absorbance of the reactant was measured with a spectrophotometer (Shimadzu UV-1800, Kyoto, Japan) at 510 nm. The total flavonoid content of the juice was expressed as mg/L rutin equivalent.

The Vc (vitamin C) content was determined through HPLC (high-performance liquid chromatography) as described by Liu et al. [13], with some modifications. The sample pre-treatment was as follows: An amount of 1 mL of juice was mixed with 5 mL 3% (*w*/*v* water) citric acid solution and left to stand for 10 min; then, the mixture was centrifuged (14,000 rpm, 4 °C, 8 min). The supernatant was passed through a 0.45 μm aqueous-phase filter membrane. The Vc content of the extract solution was analyzed through an HPLC (Water Model 6000 A; Milford, MA, USA) equipped with a UV-C detector. Separations were performed on a C18 chromatographic column (250 mm × 4.6 mm, 5 μm) using acetonitrile/water (70:30) with 0.01 M NH_4_H_2_PO_4_ (adjusted to pH 4.3 with orthophosphoric acid) as the mobile phase. The detection parameters were as follows: column temperature, 30 °C; flow rate, 0.6 mL/min; detection wavelength, 210 nm; injection volume, 10 μL. The results were calculated based on the standard curve plotted for ascorbic acid.

In accordance with the method described by Lim et al. [14], 0.05 mL juice and 3.9 mL DPPH (2,2-diphenyl-1-picrylhydrazyl) (60 μmol/L) methanol solution were mixed evenly, and the mixture was left in the dark at 25 °C for 30 min. At the same time, 0.05 mL 80% ethanol was added to 3.9 mL DPPH as the blank control. The absorbance was measured with a UV–Vis spectrophotometer (Shimadzu UV-1800, Kyoto, Japan) at 517 nm. The DPPH radical scavenging rate (%) = [1 − A1/A0] × 100, where A0 is the absorbance of the blank control, and A1 is the absorbance of the sample.

The ferric-reducing antioxidant power (FRAP) of the juice was determined according to the method of Benzie and Strain [15]. The FRAP reagent was freshly prepared in the dark and consisted of 2,4,6-tripyridyl-s-triazine (10 mM in 40 mM HCl), FeCl_3_ (20 mM), and acetate buffer (0.3 M, pH 3.6). Thereafter, 50 µL of juice was added to 2.85 mL FRAP reagent, and the mixture was kept in the dark at 37 °C for 30 min and subsequently cooled to room temperature. The absorbance was measured with a spectrophotometer (Shimadzu UV-1800, Kyoto, Japan) at 593 nm. The result was expressed as μmolFeSO_4_7H_2_O/mL of juice.

### 2.5. Secondary Metabolites

The juice was freeze-dried and extracted in 70% methanol at 4 °C for 12 h. The extract was centrifuged (12,000 rpm, 4 °C, 10 min). Thereafter, the supernatant was filtered through a 0.22 μm organic-phase filter membrane and stored in an injection bottle at –80 °C. The secondary metabolites of the celery juice were analyzed with an LC-ESI-MS/MS system (HPLC, Shimadzu Nexera X2; MS, Applied Biosystems 4500 QTRAP) using an Agilent SB-C18 Column (1.8 µm, 2.1 mm × 100 mm). Water (0.1% methanol) and acetonitrile (0.1% formic acid) were used as solvents A and B, respectively. The gradient program was as follows: the B phase ratio was 5% at 0.00 min; then, phase B increased linearly to 95% within 9.00 min and was maintained at 95% for 1 min; subsequently, phase B decreased to 5% between 10.00 min and 11.10 min, and finally, phase B equilibrated at 5% at 14 min. Linear ion trap (LIT) and triple quadrupole (QQQ) scans were performed using a triple quadrupole linear ion trap mass spectrometer (Q TRAP), AB4500 Q TRAP UPLC/MS/MS system, equipped with an ESI Turbo ion spray interface controlled through Analyst 1.6.3 software (AB Sciex). The operating parameters of the ESI source were as follows: ion source, turbine spray; source temperature, 550 °C; ion spray voltage, 5500 V (positive ion mode)/–4500 V (anion mode); ion source gas I, gas II, and curtain gas were set to 50, 60, and 25.0 psi, respectively; and collision-induced ionization parameters were set to high. Instrument tuning and quality calibration were performed with 10 and 100 μmol/L propylene glycol solutions in QQQ and LIT modes, respectively. QQQ scans used MRM mode and set the collision gas (nitrogen) to medium. The DP and CE of the various MRM ion pairs were completed by further DP and CE optimization. A specific set of MRM ion pairs was monitored at each period based on the metabolites eluted within each period.

### 2.6. Volatile Flavor Compounds

The volatile metabolites of the celery juice were assayed by automated headspace solid-phase microextraction (HS-SPME) combined with GC/MS (8890 GC, 5977B MS; Agilent Technologies, Santa Clara, CA, USA). The juice (2 mL) was placed into an SPME glass vial containing 10 μL (50 μg/mL) NaCl as an internal standard. The vial was equilibrated at 100 °C for 5 min. The fiber was inserted into the vial promptly for headspace extraction for 15 min. Thereafter, the vial was connected to the injection port and desorbed for 5 min at 250 °C. Helium was used as the carrier gas at a flow rate of 1.2 mL/min. The chromatographic column was a DB-5MS capillary column (30 m × 0.25 mm × 0.25 μm, Agilent J&W Scientific, Folsom, CA, USA). The column temperature program was 40 °C for 3.5 min, 100 °C at a 10 °C/min rate, then 180 °C at a 7 °C/min rate, and finally 280 °C at a 25 °C/min rate, which was held for 5 min. The mass spectra conditions were as follows: mass spectrometry interface temperature, 280 °C; ionization energy, 70 eV; and scan range from 50 to 500 m/z. Volatile compounds were identified by comparing each mass spectrum with spectra from the NIST Standard Reference Database Number 69 at a quality match score of greater than 85%, and the retention index (RI) of each volatile compound was calculated using a series of n-alkanes (C7–C30).

### 2.7. Statistical Analysis

The software package SPSS 25 was used for the statistical analysis. One-way ANOVA (Duncan’s test, significance level 0.05) was used to analyze the differences in physical and chemical properties among juices from different varieties. Principal component analysis (PCA) was conducted to evaluate the function of celery juice from different varieties. Data were expressed as the mean ± standard deviation of triplicate samples.

## 3. Results and Discussion

### 3.1. Basic Physical and Chemical Properties

The physical and chemical properties of the juices of 26 celery cultivars are shown in Tables S1 and S2. Single plant weight and juice yield are the indexes reflecting the celery juice extraction efficiency. Among the 26 celery cultivars, the single plant weight ranged from 0.301 to 1.294 kg, and the variation coefficient was 47.6%. CL8, CL10, CL14, CL18, CL21, and CL25 had a high plant weight (>1 kg), while six other cultivars had a low plant weight (<0.4 kg). These small plants were all Chinese celery. The highest juice yield was 83.9% (in CL7), followed by 79.6% (in CL10), 78% (in CL18), and 76.1% (in CL19). Buttkus [16] reported that the juice yield of the celery leaf is 70–80%, which is consistent with the results of this study. There was a significant correlation between single plant weight and juice yield (*p* < 0.01) (Table S3). Therefore, considering the juice making efficiency, Chinese celery is not suitable.

**Figure 2:**
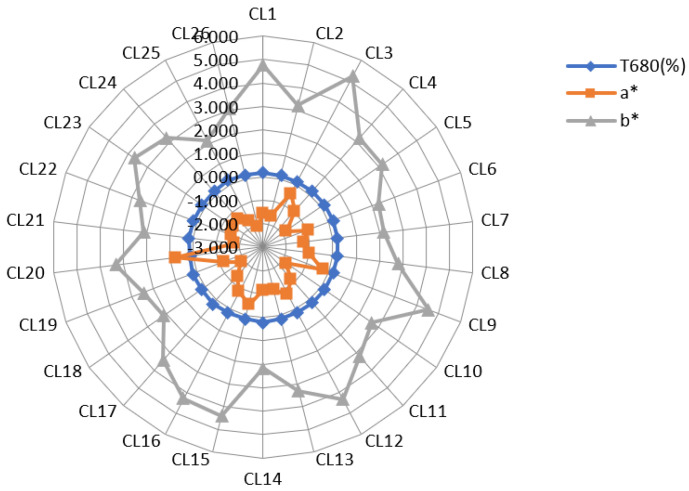
The T680 (%), a* value, b* value of the juices of 26 celery cultivars.

Color is an important evaluation index affecting the appearance of fruit and vegetable juices. Colorimeters have been widely used to detect the color changes among different juices, such as pomegranate [17], apple [18], and mango [19]. In this study, the variation coefficients for the L* value and transmittance (T680) were 8.8% and 13.9%, respectively. The variation coefficients for the a* and b* values were 51.6% and 27.9%, respectively (Figure 2). Therefore, the color difference among the juices mainly depends on the a* and b* values. Most juices were chartreuse to light green. CL3 and CL15 have white petiole, CL9 and CL20 have red petiole. The juices color of these four cultivars were significant different among the 26 juices. SSC and soluble sugar content can influence the taste of fruit and vegetable juice [20]. In this study, the SSC of the juice was positively correlated with the soluble sugar content (*p* < 0.01) (Table S3). The SSC and soluble sugar content ranged from 3.333% (in CL12) to 8.433% (in CL5) and 1.09 mg/mL (in CL3) to 5.121 mg/mL (in CL5), respectively. The highest soluble sugar content was 5.121 mg/mL (in CL5), followed by 4.582 mg/mL (in CL18), 4.381 mg/mL (in CL19), and 4.087 mg/mL (in CL26). Therefore, the juice of these four cultivars may taste sweeter than the other cultivars. Mezeyova et al. [21] found a significant difference in the SSC content among six celery cultivars. Soluble dietary fiber (SDF) can improve glycemic regulation and intestinal health [22]. Our results show that there were significant differences in the SDF content among the juices of the 26 celery cultivars, and its variation coefficient was 8.6% (Table S1).

Celery contains abundant flavonoids and other phenolic compounds and has strong antioxidant characteristics [23,24]. Flavonoids, total phenolic compounds, Vc contents, the DPPH inhibition rate, and FRAP are important indexes to evaluate the antioxidant capacity of celery juice (Table S2). The variation coefficient of the flavonoid content in the different celery cultivars’ juices was 24.4%. The highest total flavonoid content was found in the CL2 juice (81.52 mg/L), while the total flavonoid content in the juices of five cultivars was lower than 40 mg/L. The variation coefficient of total phenolic compounds (17.6%) and Vc (15.3%) contents was lower than that of the flavonoid content. The total phenolic compound content ranged from 158.28 mL/L (in CL18) to 315.54 mL/L (in CL26). The Vc content varied between 68.63 mg/L (in CL26) and 40.68 mg/L (in CL18). The highest DPPH inhibition rate and FRAP were found in the CL2 juice, which had the highest flavonoid content. In contrast, seven varieties’ juices (CL3, CL6, CL8, CL13, CL14, CL22, and CL25), with a low DPPH inhibition rate or FRAP, also had a low flavonoid content. Liu reported [25] that flavonoids make the highest contribution to the DPPH-scavenging activity of celery juice. FRAP and DPPH were significantly positively correlated with total flavonoids and total phenolic compounds, indicating that total flavonoid and total phenolic compound contents can reflect the antioxidant capacity of celery juice (Table S3). Yao et al. [26] found that the antioxidant capacity in celery was positively correlated with flavonoids, total phenolic acids, and total phenolic compounds.

### 3.2. Principal Component Analysis (PCA)

The PCA results show that the cumulative variance contribution rate was 80.322%. The variance contribution rate of the first principal component (PC1) was 30.626%, which can reflect the antioxidant capacity of the celery juice. The variance contribution rate of the second principal component (PC2) was 22.429%, which can reflect the appearance of the celery juice and the juice-making efficiency of different celery cultivars. The variance contribution rate of the third principal component (PC3) was 17.687%, which has an obvious positive correlation with the SSC and soluble sugar content. According to the PC scores (Table S4), the juices of CL5, CL6, CL8, CL19, and CL26 may taste sweeter than the other cultivars, and the juices of CL1, CL2, and CL26 had a higher antioxidant capacity. Shahkoomahally et al. [17] found that the physical and chemical properties of pomegranate juice were significantly different among fourteen genotypes. Based on 18 quality characteristics, Li et al. [27] screened six citrus cultivars suitable for whole fruit juice production. Liu et al. [20] obtained a sweet potato variety suitable for juice making from 15 sweet potato cultivars. Although many reports have analyzed the juice-making properties of different fruit cultivars, the research on celery juice mainly focuses on the juice-making technology and its health function. This study analyzed celery cultivars suitable for juice making, which can provide a reference for celery juice production and can guide vegetable juice making at home.

### 3.3. Secondary Metabolites

**Figure 3:**
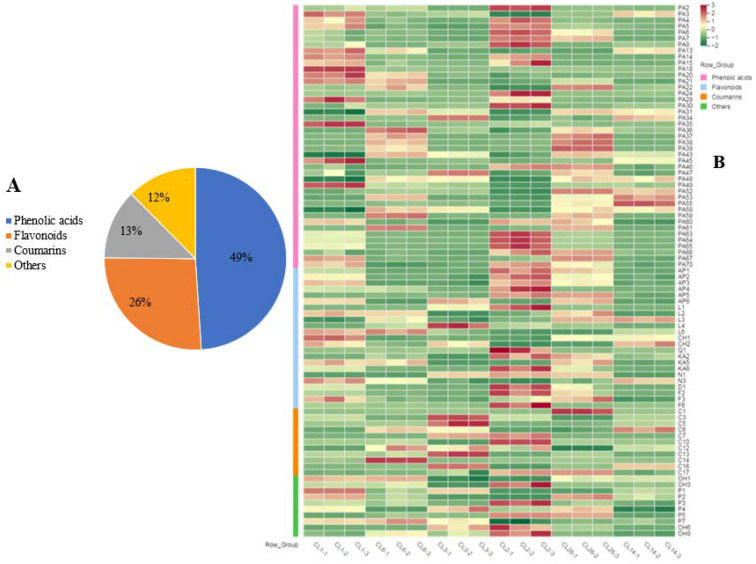
The classification of secondary metabolites (**A**) and the heatmaps of relative content of different secondary metabolites (**B**) in celery juice. All the relative contents normalized using z-score. The abbreviations of different volatile flavor compounds were show in Table S6.

The PCA results show that the first principal component was significantly correlated with phenolic compounds, flavonoids, and antioxidant capacity in the celery juice, which affected the health quality of the juice. Therefore, the juices of CL1, CL2, and CL26 with a high score for PC1 (HJ) and the juices of CL6, CL3, and CL14 with a low score (LJ) were selected. The secondary metabolites in the six cultivars of celery juice were analyzed through LC/MS. A total of 145 secondary metabolites were obtained (Table S5), including 71 phenolic acids, 38 flavonoids, 18 coumarins, and 7 phthalides (Figure 3A), of which 87 had significant differences in the six celery juices (Table S6). These results show that celery juice is rich in secondary metabolites, and there are significant differences in the content of secondary metabolites among different cultivars. The same conclusion was made regarding pomegranate [28], feijoa [29], and broccoli sprout [30] juices.

**Figure 4:**
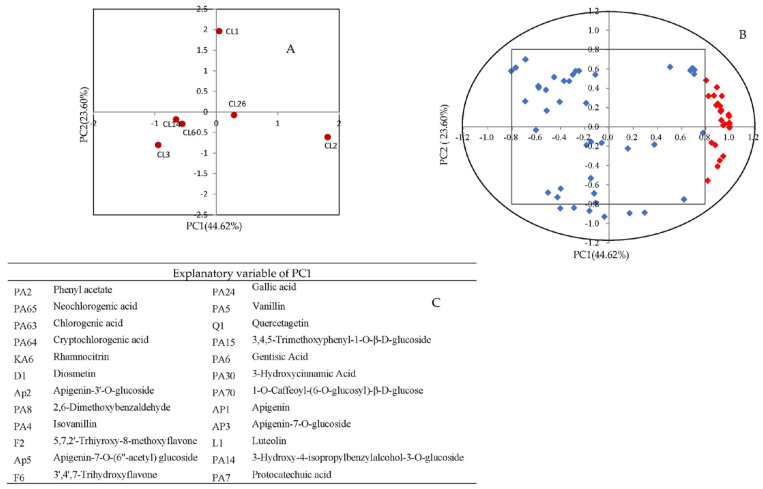
Principal component analysis of juice made from six celery cultivars (CL1, CL2, CL26, CL6, CL3, and CL14) using 43 phenolic acids and 23 flavonoids as variables. (**A**) Projection of cultivars; (**B**) distribution of variables; (**C**) the explanatory variables (14 phenolic acids and 10 flavonoids) of PC1 (red diamond, load value > 0.8).

**Figure 5:**
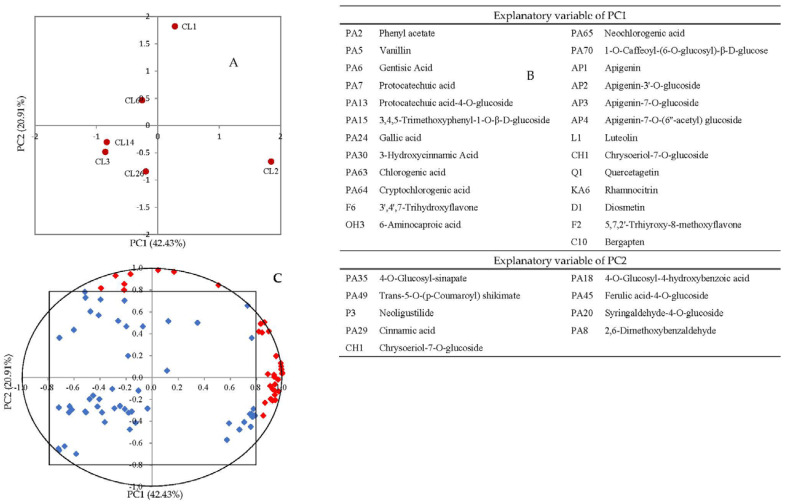
Principal component analysis of juice made from six celery cultivars (CL1, CL2, CL26, CL6, CL3, and CL14) using 87 different metabolites as variables. (**A**) Projection of cultivars; (**B**) distribution of variables; (**C**) the explanatory variables (26 compounds) of PC1 and the explanatory variables (seven compounds) of PC2 (red diamond, load value > 0.8).

The PCA of 66 phenolic compounds, including 43 phenolic acids and 23 flavonoids, showed that the vertical axis can separate six samples from the antioxidant capacity (Figure 4A), which further confirmed the results of basic properties analysis in the juices of different celery cultivars. Each PC selects the index whose load value is greater than 0.8 as the explanatory variable. Load value of 14 phenolic acids and 10 flavonoids were higher than 0.8 (Figure 4B,C) and were positive correlated with the antioxidant capacity of celery juice. However, the PCA of 87 different metabolites show that HJ and LJ cultivars cannot be separated from the antioxidant capacity. The cumulative variance contribution rate of the first two PC is 63.34% (Figure 5A), which can reflect most information. The variance contribution rate of PC1 was 42.43%, the explanatory variable (load value > 0.8) including 13 phenolic acids, 10 flavonoids, bergapten, 6-aminocaproic acid, and neoligustilide. The variance contribution rate of PC2 was 20.91%, the explanatory variable (load value > 0.8) including seven phenolic acids and one flavonoid (Figure 5B). Compared with other five cultivars, the juice of CL2 has higher content of most explanatory variables. These results indicated that the juice of CL2 has higher content of secondary metabolites.

Flavonoids act as free-radical scavengers and antioxidants, exhibiting antimutagenic, anti-inflammatory, and antiviral effects [31]. In this study, 22 flavones, including apigenin and its derivatives, luteolin and its derivatives, and chrysoeriol and its derivatives; 8 flavonols, mainly including kaempferol and its derivatives; and 8 flavanones, mainly including naringin and its derivatives, and diosmetin and its derivatives, were detected. The content of 23 flavonoids showed significant differences in the juices of the six celery cultivars (Figure 3B). The contents of 14, 15, and 14 flavonoids, respectively, in the CL1(HJ), CL2(HJ), and CL26(HJ) juices were more than twice those found in the juice from the cultivar with the lowest content. Compared with HJ cultivar juices, most flavonoid contents in the LJ cultivar juices were the lowest. Our previous research showed that there were significant differences in the flavonoid content among different celery accessions [32]. Moreover, the contents of apigenin and its derivatives in the HJ cultivar juices were higher than those in the juices of the cultivar with the lowest content (Figure 3B). The main flavonoid in celery is apigenin, which has been used as a traditional or alternative medicine for centuries. It has numerous therapeutic functions for diabetes, amnesia, Alzheimer’s disease, depression, insomnia, and cancer [33]. Compared with other structurally related flavonoids, apigenin has low intrinsic toxicity and can strike cancer cells accurately [34]. So, we conjecture that the different of apigenin and its derivatives responsible for the different of flavonoids in celery juice.

Phenolic acids are classified into hydroxybenzoic and hydroxycinnamic acids, which are linked to several health benefits [35]. Chlorogenic acid can induce cancer cell differentiation to effectively treat cancers [36]. It was reported that the main phenolic acids in celery are chlorogenic acid, caffeic acid, p-coumaric, and ferulic acids [25]. A total of 71 phenolic acids were determined in this study, including 27 hydroxybenzoic acids and their derivatives, and 44 hydroxycinnamic acids and their derivatives. There were significant differences in the contents of 43 phenolic acids among different cultivars’ juices (Figure 3B). The maximum content differences in chlorogenic acid, protocatechuic acids, caffeic acid, ferulicacid, gallic acid, and sinapoyl glucuronic acid were more than 10-fold. Compared with the juices of five other celery cultivars, most phenolic acid contents in the CL3 juice were the lowest.

Coumarins are common in many vegetables and fruits. These compounds can not only improve plant growth and health but are also useful in the pharmacotherapy of cancer [37]. In previous studies, four coumarins (bergapten, psoralen, xanthotoxin, and isopimpinellin) were detected in celery [38]. In this study, 18 coumarins were detected, 11 of which were significantly different in the juices of the six celery cultivars (Figure 5B). The maximum content differences in psoralen, isoscopoletin, and scopolentin were more than 5-fold (Table S6). Among the juices of the six celery cultivars, the contents of five coumarins were the highest in the CL3 juice (Figure 5B). Due to the light-sensitive properties of psoralens, high concentrations of psoralen intake may cause phytophotodermatitis; psoralens can also alter the pharmaco-kinetics of co-ingested drugs [39]. Therefore, CL3 is not recommended to make juice for health reasons.

In addition, 18 other secondary metabolites were detected, including seven phthalides. Phthalides are naturally bioactive compounds, with many health benefits, and are particularly abundant in plants from the Apiaceae family. Phthalides can be determined through HPLC, LC/MS, or GC/MS. A total of 71 phthalides and their derivatives, as well as their associated dimers, have been identified in Apiaceae plants and four plants from other families [2]. In an overview of celery aroma substances, 12 phthalides were found in 15 reports [40].

### 3.4. Volatile Flavor Compounds

**Figure 6:**
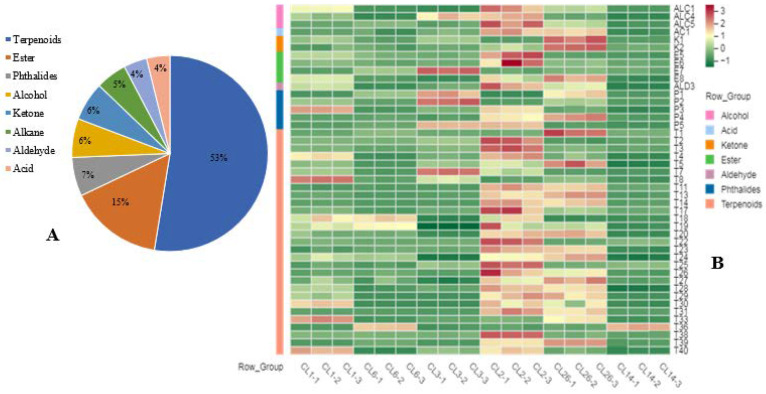
The classification of volatile compounds (**A**) and the heat map of relative content of different volatile flavor compounds (**B**) in celery juice. All the relative contents normalized using z-score. The abbreviations of different volatile flavor compounds were show in Table S8.

Aroma is an important sensory property of celery juice. The volatile compounds in the juices of the six above-mentioned cultivars were analyzed through GC/MS. A total of 78 volatile compounds were obtained in the celery juices (Table S7), including five alcohols, three acids, five ketones, three aldehydes, four alkanes, five phthalides, twelve esters, and forty-one terpenoids (Figure 6A), of which forty-five were significantly different among the six celery cultivars (Table S8). The differential volatile compounds included twenty-nine terpenoids, four esters, three alcohols, one acid, one aldehyde, two ketones, and five phthalides (Figure 6B). Turner et al. [41,42,43] found that the volatile compounds of eight celery cultivars from the United States, United Kingdom, and several European countries were similar, but the planting environment, climate, and harvest time significantly affected the content of volatile compounds in celery. The types of volatile compounds are different between the detected results in this study and those in the study by Turner. These differences may be due to the different cultivars of celery juice (including celery cultivars from China) used in this experiment. In addition, the instability of volatile compounds and the differences in the determination methods also lead to differences in the results.

**Figure 7:**
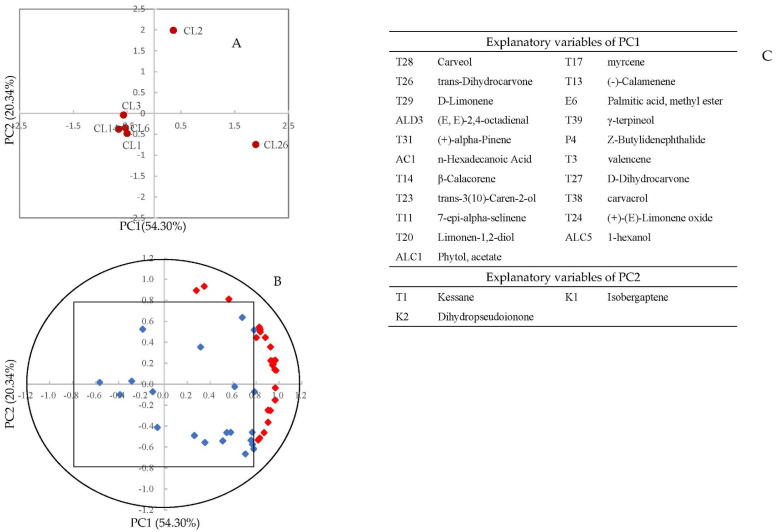
Principal component analysis of juice made from six celery cultivars (CL1, CL2, CL26, CL6, CL3, and CL14) using 45 differential volatile compounds as variables. (**A**) Projection of cultivars; (**B**) distribution of variables; (**C**) the explanatory variable (22 compounds) of PC1 and explanatory variable (three compounds) of PC2 (red diamond, load value > 0.8).

The PCA of 45 differential volatile compounds showed that the juices of CL1, CL3, CL6, CL14 were assembled and were separated with CL2 and CL26 by the vertical axis (Figure 7A). The CL2 and CL26 were separated by horizontal axis. The variance contribution rate of PC1 was 50.30%, the explanatory variable including 16 terpenoids, two alcohols, n-hexadecanoic acid, palmitic acid, methyl ester, and (E, E)-2,4-octadienal. The variance contribution rate of PC2 was 20.34%, the explanatory variable, including isobergaptene, dihydropseudoionone, and 3-n-butylphthalide (Figure 7B,C). So, we speculate that the juices of CL1, CL3, CL6, and CL14 may have similar odor. The unique odor of celery is due to phthalides [2]. Therefore, the juices of CL2 and CL26 may have strong celery odor.

The contents of 31 and 35 volatile compounds, respectively, in the CL26 and CL2 juices were more than twice those found in the juice from the cultivar with the lowest content (Figure 6B). The maximum content difference in 3-n-butylphthalide among the six cultivars of celery juice was 14.81-fold. The maximum content difference in D-limonene, neocnidilide, and β-(E)-ocimene was more than 10-fold. Terpenoids, including limonene, ocimene, terpinene, pinene, and selinene, which constitute the main celery flavor (fresh, herbal, woody, and citrusy), are the most abundant volatile compounds in celery [40]. These terpenoids were also detected in this study and showed significant content differences among the juices of the six cultivars. The juices of CL1, CL2, and CL26 (from China) had a higher terpenoid content than the CL6 (from America) juice and the CL14 (from the Netherlands) juice. In this study, eleven phthalides were detected in the celery juice using LC/MS and GC/MS. The contents of ten phthalides were significantly different in the juices of the six celery cultivars. Phthalides have various medicinal functions, such as promoting cancer cell apoptosis, inhibiting atherosclerosis, regulating thrombosis, and providing protection against cerebral ischemia and hypertension [44,45,46]. Lu et al. [47] found that 3-butylphthalide can be used as a functional and natural supplement for the treatment of obesity and related diseases.

## 4. Conclusions

According to the literature mentioned in the introduction, celery juice benefits people’s health. In this study, we evaluated nine basic physical and chemical indexes and five antioxidant capacity indexes of the juices made from 26 celery cultivars. Then, the bioactive constituents in juices from six celery cultivars were analyzed by LC/MS and GC/MS. These results showed that the color, juice yield, antioxidant capacity, and the content of soluble sugar were significantly different among celery juices from different cultivars. There were many bioactive constituents in celery juice, and some of them have great differences in the juices from different celery cultivars. So, celery juice may have a great potential to improve people’s health. Compared with other cultivars, the juices from Chinese celery cultivars have more bioactive constituents, while the juices from the United States and European celery cultivars have higher juice yield. The bioactive constituents found in this study could be used to analyzing the health functions of celery juice, meanwhile the evaluation results could guide celery production and breeding.

## Figures and Tables

**Table 1:** The names and origins of 26 celery cultivars.

Number	Variety Name	Origin
CL1	Huangxin Qin	Shanghai, China
CL2	Tie gan qin cai	Hebei, China
CL3	White petiole Qin	Tianjin, China
CL4	Jin nan shi qin No. 1	Tianjin, China
CL5	Si ji qing xiang little Qin	Tianjin, China
CL6	Ventura	America
CL7	Tango	Netherlands
CL8	Queen	France
CL9	Shen Qin No.2	Shanghai, China
CL10	Betty	America
CL11	Fast-growing celery	Japan
CL12	Golden Plume 4162	America
CL13	Hong cheng hollow Qin	Tianjin, China
CL14	Dutch giant celery	Netherlands
CL15	Xue bai qin cai	Sichuan, China
CL16	Yellow seedling Qin	Tianjin, China
CL17	Huang nen celery	France
CL18	California King	America
CL19	Bo li cui shi qin	Henan, China
CL20	Purple petiole Qin	Shanxi, China
CL21	Emperor	France
CL22	Summer celery	Italy
CL23	Jin nan shi qin No. 3	Tianjin, China
CL24	Shang nong yu qin	Shanghai, China
CL25	Tall Utah 52–70 R	America
CL26	Zhang qiu Bao Qin	Shandong, China

## Data Availability

Not applicable.

## Supplementary Materials

The following supporting information can be downloaded at: https://www.mdpi.com/article/10.3390/foods11182719/s1, Table S1: The basic physical and chemical indexes of the juices of 26 celery cultivars; Table S2: The antioxidant capacity indexes of the juices of 26 celery cultivars; Table S3: Pearson’s correlation coefficients among 14 indexes of the juice of 26 celery cultivars; Table S4: Principal components score of different cultivars celery juice; Table S5: Secondary metabolites of celery juice; Table S6: The relative content of secondary metabolites in the celery juice of six cultivars; Table S7: Volatile compounds of celery juice; Table S8: The relative content of volatile compounds in the celery juices of six cultivars.
